# Smoking, health and ageing

**DOI:** 10.1186/1742-4933-5-10

**Published:** 2008-09-16

**Authors:** Vittorio Nicita-Mauro, Giorgio Basile, Giuseppe Maltese, Claudio Nicita-Mauro, Sebastiano Gangemi, Calogero Caruso

**Affiliations:** 1 Chair of Geriatrics and Gerontology, University of Messina, Messina, Italy; 2 Chair of Allergy and Clinical Immunology, University of Messina, Messina, Italy; 3 Immunosenescence Unit, University of Palermo, Palermo, Italy

## Abstract

On March 19, 2008 a Symposium on Pathophysiology of Ageing and Age-Related diseases was held in Palermo, Italy. Here, the lecture of V. Nicita-Mauro on Smoking, health and ageing is summarized. Smoking represents an important ageing accelerator, both directly by triggering an inflammatory responses, and indirectly by favoring the occurrence of several diseases where smoking is a recognized risk factor. Hence, non-smokers can delay the appearance of diseases and of ageing process, so attaining longevity.

## Background

On March 19, 2008 a Symposium on Pathophysiology of Ageing and Age-Related diseases was held in Palermo, Italy. Here, the lecture of V. Nicita-Mauro on Smoking, health and ageing is summarized.

**Figure 1:**
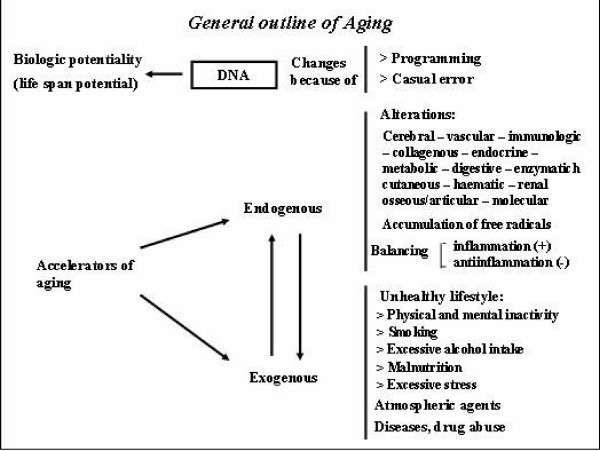
**General outline of ageing. **Smoking represents an important exogenous accelerator of ageing process.

Ageing is a very complex biological phenomenon deriving from an interaction between genetic and environmental factors [1]. Among these latter, the smoke of cigarettes represents an important accelerator of the ageing process (Figure 1), both directly through complex mechanisms mediated prevalently by excessive formation of free radicals, and indirectly by favouring the appearance of various pathologies in which smoke is a recognized risk factor [2].

Oxidative stress is a possible mechanism consistent with dangerous effects of smoking since cigarette smoke contains free radicals that activate inflammatory cells with inflammatory mediator production and further oxidative damage [3,4]. Smokers have significantly lower erythrocyte CuZn SOD and Se-GSH-Px activities than non-smokers [5]. Accordingly, concentrations of serum antioxidant vitamins, such as ascorbic acid and vitamin E, have been reported to be lower in chronic smokers than in non-smokers [6]. Hence, these findings support the concept that smoking is associated with an increased oxidative stress that may cause and/or accelerate age-related inflammatory diseases [7].

Telomere length represents another possible connection between smoking and ageing. Telomere length shortens with age in all replicating somatic cells, and it has been shown that tobacco smoking enhances telomere shortening in circulating lymphocytes [8], hence worsening the immunosenescence state characteristics of ageing [9].

There is no doubt that smoke is an important risk factor for many diseases, in particular for cardiovascular, neoplastic and respiratory diseases, which are the main causes of death in the industrialized Countries, where smoking habit is also largely diffuse. According to the projections, by the year 2020, smoking will become the main cause of death and disability, with more than 10 million death cases per year [2].

### Smoking and age-related diseases

**Figure 2:**
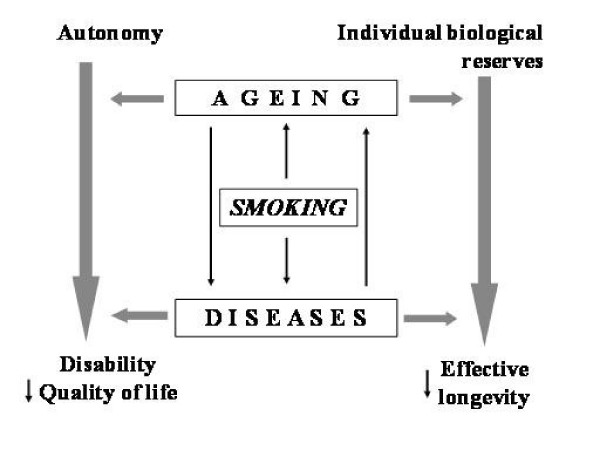
Cigarette smoking is an important risk factor for many diseases and an accelerator of ageing, it compromises not only life expectancy but also the quality of life.

Smoking plays an important role also in the development of other pathological conditions being particularly frequent in old age, such as dementia, osteoporosis, diabetes, peptic ulcer, gastro-esophageal reflux, erectile dysfunction, senile macular degeneration, nuclear cataract, alterations of hearing and skin. Smoke compromises not only life expectancy, but also the quality of the life, favouring the occurrence of non-autonomy (Figure 2) [2].

The role of smoking in dementia and Alzheimer's disease (AD) has been debated. In the last years, several case-control studies suggested that smoking was associated with decreased risk of dementia [7,10]. On the other hand, it has been hypothesized that findings obtained in case-control studies were a consequence of survival bias rather than a true protective effect of smoking [11]. However, the mechanisms by which smoking would prevent the risk of AD should be related to the positive nicotinic effects of smoking on cognitive functioning [7,12]. On the other hand, the increased frequency of cardiovascular and cerebrovascular illnesses among smokers is likely to increase the risk of AD in later life [13,14]. Thus, the association between smoking and risk of dementia, including AD, is debated. However, the results of a recent meta-analysis of prospective studies clearly showed that, when compared with people who have never smoked, current smokers have an increased risk of dementia and cognitive decline ranging from 40 percent to 80 percent, depending on the outcome examined [10].

As regards the relationships between smoking and cardiovascular system, several studies have shown that smokers have higher average systolic blood pressure values, compared to non-smokers, further, smokers do not have the physiological nocturnal decrease of the blood pressure levels [2]. However, the after-smoking increase of the arterial blood pressure can be abolished by a preventive application of the calcium-antagonist nifedipin [15]. The smoke is able to cause tissue oxidative damage at various levels, and contributes significantly to the appearance of endothelial dysfunctions, and to the alterations which induce the arteriosclerotic process. It is known that in smoker subjects the oxidative stress derives from excessive increase of free radicals and reduction of the antioxidant mechanisms (enzymatic and non-enzymatic type). It has been shown that the alteration of endothelial vasoregulatory activity is independent from the number of smoked cigarettes, as this activity was similar in subjects who smoked one package of cigarettes per week or per day (or less) [2].

A lot of epidemiologic study support the role of smoke in acute coronary syndrome (ACS) and myocardial infarction (MI). It is noteworthy that the MI incidence of in Britain has fallen markedly in recent years. The British Regional Heart Study examined changes in cardiovascular risk factors and MI incidence over 25 years from 1978 in a cohort of 7735 men. The hazard of MI decreased of a 62% decline over the 25 years. Forty-six percent of this decrease in MI hazard could be explained by a combination of changes in the major risk factors over this time: a fall in the number of cigarette smokers was the most powerful of all [16]. Furthermore, in a follow up of 54 783 women and men from the prospective Danish Diet, Cancer and Health study who were 50 to 64 years at baseline and free of coronary artery disease, during a median of 7.7 years, 1127 incident cases of occurred. Obesity conferred an elevated risk of ACS in both healthy and less healthy subgroups of lifestyle behaviors: however, the authors found that obese individuals had a considerable lower risk if they were nonsmokers [17].

Non-smokers have a much higher life expectancy than smokers[18], and the suspension of smoking is accompanied, even in the elderly, by an increase in the survival time due to the reduction of smoke-induced biological damage. Therefore, cigarette smoking is opposing the longevity, particularly the extreme one, as it is confirmed by the observations obtained on centenarians [2]. Tobacco smoking is associated with a compromised health status and impaired autonomy also in the centenarians.

Modifiable healthy behaviours during early elderly years, including smoking abstinence, weight management, blood pressure control, and regular exercise, are associated not only with enhanced life span in men but also with good health and function during old age [19].

Considering the demonstrated beneficial effects of suspension of smoking, all practitioners and geriatricians in particular, should promote the abstinence from smoking as a behavioural norm for a correct life style.

Therefore, it is our duty to notify non smokers to keep non-smoking, and to the smokers to abandon smoking, evidencing the risks related to smoking, and the limitations to health deriving from this habit. On the other hand, we have to illustrate also the better performance that non-smokers may achieve. The invitation by the practitioner to stop smoking may be effective, but often this possibility is not utilized properly, in particular as far as elderly subjects is concerned: indeed these subjects often were never been told to leave smoking [20].

## Conclusions: Stopping Smoking

The first requirement of stopping smoking is certainly the motivation of the smoker himself to do this, since without this motivation any attempt is vane. Today, numerous strategies exist, either of pharmacological or non-pharmacological type, which are advantageous also for the elderly. The most effective smoking cessation programs involve a combination of pharmacotherapy and behavioural and/or cognitive counselling to improve abstinence rates.

Approved pharmacological treatments include nicotine replacement therapies, bupropion, a non nicotine option, antidepressants, drugs targeting cannabinoid receptors and newer pharmacological approaches including the selective nicotinic partial agonists [21-26].

Nicotine can be applied in various ways, but the best is certainly the transdermic method through the application of patches. The efficacy of the nicotine patches has widely been documented in a series of studies, together with the contraindications, the side effects, the pharmacological interactions, even for the elderly subjects [20,21]. Another method is the use of an antidepressant, called bupropione, which has demonstrated good efficacy as both a monotherapy, and in association with nicotine patches [22].

Varenicline, an alpha4beta2 nicotinic acetylcholine receptor partial agonist, is the most recent agent approved for smoking cessation. This drug works by reducing the strength of the smoker's urge to smoke and by relieving withdrawal symptoms. Varenicline mimics the effect of nicotine and hence reduces craving when smokers stop. Furthermore, varenicline blocks nicotine receptors and in this manner, provokes a weaker response to nicotine if smokers use tobacco products while taking the drug. Recent evidence suggests that the nicotin-receptor partial agonist varenicline is at least as effective as nicotine replacement therapy and antidepressants [23-26].

## Competing interests

The authors declare that they have no competing interests.

## Authors' contributions

All authors contributed equally to the paper and read and approved the final manuscript.
